# The Medico-Social Aspects of the Consumption Problem

**Published:** 1913-04-05

**Authors:** 


### THE MEDICO-SOCIAL ASPECTS OF THE CONSUMPTION PROBLEM.

### I.—Ideals and Possibilities.

Mr. Lloyd George has done his best to advertise the ideals which have animated the Government in approaching the question of the treatment of consumption under the National Insurance Act, and there can be no question that many of the supporters of the Act, Mr. Lloyd George not least of all, firmly believe, and honestly, that the National Insurance Act is the best solution of the problem how to deal with consumption as it affects the community. But it must be remembered that those who drew up the Act and who were responsible for its clauses know comparatively little about the expert views which are held with regard to this very important matter. It is the moral and practical duty of the profession and the public to scrutinise most closely the suggestions which are being made, and which are being countenanced by the Government under the National Insurance Act, for the general fight against the disease. At the present moment the public, there can be little question, is in a state of panic with regard to consumption. So persistently and so vehemently have certain aspects of this problem been presented to the layman that he has lost his sense of proportion! This is not to be wondered at, for the problem is an intricate one, and the opinions with regard to it are conflicting. Where doctors agree to differ it can scarcely be reckoned culpability on the part of the public if it elects to follow the most pessimistic opinion and to exaggerate the dangers which it may incur.

### Public Aspects of the Problem.

The medico-sociological aspects of the disease are important when we consider that there is still considerable doubt as to the necessity for methods of segregation and isolation of consumptives, since we are not perfectly sure in what manner the disease is transmitted. Under this category it will be necessary to discuss such vital subjects as the meat and milk supply, the home conditions of consumptives and consumption contacts, general housing questions, and matters which fall under the domain, principally, of the medical officers of health. This side of the problem we may call the preventive side; and it is to this, admittedly, that our most active efforts must be directed. Expenditure upon improvements that may ameliorate the insanitary conditions of our large towns must be welcomed cordially by everyone who believes, as we believe, that improved hygienic conditions are the necessary preliminaries for any campaign directed against a disease, no matter how caused.

### Points of Attack.

Secondly, we shall devote ourselves to the study of the curative measures that have been proposed. This part of the problem is a much more specialised one. Yet it is a part of the problem that urgently needs criticism and discussion. Practically—and this is the arrangement we shall follow in these papers—the curative aspect may be divided into two main divisions: the treatment, on the one hand, of the consumptive in the early stages of the disease; and, on the other, the treatment of the patient with advanced disease. Each division again may be looked at from two different sides; from the point of view of the patient himself, and from that of the community; in other words, the individual and the communal view. Still further subdivision enables us to particularise a socio-economic aspect, and here again it is of the greatest importance that we shall take into consideration the facts gleaned from the experience of workers all over the world, and to argue not only from deduction but by analogy.

Hitherto, the matter has been approached, almost invariably, from the point of view of specialists. On the one side we have had the economic specialists who have insisted that the economic considerations are the most important; on the other the medical, who have similarly harped on the expert dogmas which science holds with regard to the disease. It is urgently necessary to bring these two groups into relation with each other, and to find out in how far their combined assistance may clear up certain doubtful points.

### A Policy of Pure Experiment.

The nation is on the eve of embarking on a policy of pure experiment; no one can say what may be the outcome. So far, as it seems to us, most of those who are able to criticise the suggestions made have, through diffidence or unwillingness to be looked upon as a pessimist, refrained from pointing out the obvious absurdities which some of these views, if put into practice, would entail. The public as a whole has consequently been led to believe that everything is for the best in this best of all possible schemes for the fight against consumption. The supporters of the dispensary system have advertised so well and so persistently that the arguments of those who point out the dangers and follies of this much exploited system have been entirely overlooked. This, we believe, is a mistake, and should be rectified as soon as possible. The lay authorities who are in charge of the local schemes under the Act must be made to realise the difference between ideals and possibilities. In this connection we have seen what an instructive criticism on the dispensary was offered last week in our columns by Dr. G. J. Masson Martin, who dealt with the use of tuberculin in Poor-Law infirmaries.

We need scarcely say that in this discussion we shall be grateful for any help or suggestion that our readers may give. The experience of sanatorium superintendents and hospital authorities is of the greatest value to those who have to consider the merits of various suggested ways out. Criticism directed against anything that may appear in this series will be cordially welcomed, so long as it is direct and grounded on experience, suggestion, or fact.

